# Plasma cathelicidin and longitudinal lung function in current and former smokers

**DOI:** 10.1371/journal.pone.0212628

**Published:** 2019-02-27

**Authors:** Robert M. Burkes, Jacquie Astemborski, Allison A. Lambert, Todd T. Brown, Robert A. Wise, Gregory D. Kirk, M. Bradley Drummond

**Affiliations:** 1 Division of Pulmonary Diseases and Critical Care Medicine, Department of Medicine, University of North Carolina-Chapel Hill, Chapel Hill, NC, United States of America; 2 Department of Epidemiology, Johns Hopkins University, Baltimore, MD, United States of America; 3 Division of Pulmonary and Critical Care, University of Washington, Spokane, WA, United States of America; 4 Department of Medicine, Division of Endocrinology and Metabolism, Johns Hopkins University, Baltimore, MD, United States of America; 5 Department of Medicine, Division of Pulmonary and Critical Care Medicine, Johns Hopkins University, Baltimore, MD, United States of America; 6 Department of Medicine, Division of Infectious Diseases, Johns Hopkins University, Baltimore, MD, United States of America; University of Pittsburgh, UNITED STATES

## Abstract

**Introduction:**

Cathelicidin (also known as LL-37 in humans) is an antimicrobial peptide secreted by epithelial and immune cells and regulated by vitamin D. The immunological roles of cathelicidin make it a putative biomarker to identify individuals at risk for reduced lung function. The objective of this study is to determine potential independent associations between low plasma cathelicidin and longitudinal lung function in current or former smokers without COPD.

**Methods:**

In a nested analysis of 308 participants from an observational cohort study, plasma cathelicidin and serum 25-hydroxy-vitamin D measurements were obtained at baseline, years three and five. The independent association between lowest quartile cathelicidin (<35 ng/ml) and forced-expiratory-volume-in-1-second (FEV1) at baseline, six and 18 months from each cathelicidin measurement was assessed with generalized estimating equations after adjusting for age, sex, race, smoking status and intensity. The long-term stability of cathelicidin and relationship with vitamin D was evaluated.

**Results:**

The cohort was 91% African-American, mean age 48.6 years, 32% female, and 81% current smokers. Participants with low cathelicidin were more likely to be female and have lower FEV1. Low cathelicidin was not independently associated with baseline FEV1. There was an independent association between low cathelicidin and reduced FEV1 at six months [-72 ml (95% CI, -140 to -8ml); p = 0.027] and 18 months [-103 ml (95% CI, -180 to -27 ml); p = 0.007]. Cathelicidin was stable over time and not correlated with vitamin D level.

**Conclusion:**

In current and former smokers with preserved lung function, low cathelicidin is associated with sustained lung function reductions at six and 18 months, suggesting that cathelicidin may be an informative biomarker to predict persistent lung function disparities among at-risk individuals.

## Introduction

The identification of current and former smokers at risk for reduced lung function over time and subsequent development of chronic obstructive pulmonary disease (COPD) is of emerging importance [1, 2]. The progression to early COPD is determined by multiple factors including *in utero* smoke exposure, active and passive smoking as an adolescent, childhood infections, low expiratory volumes at younger ages, and the presence of asthma as a child [3–8]. Importantly, frequent airway infection and propensity to develop pneumonia have a contribution to lung function impairment and subsequent COPD development [4, 9]. Determination of the impact of deranged host immunity on lung function impairment and identification of measurable clinical markers of pulmonary innate immune function holds prognostic and therapeutic importance.

Cathelicidin (also known as LL-37 in humans) is an antimicrobial peptide secreted by airway epithelium and immune cells that has broad immunologic functions including direct microbial killing, immune cell signaling, lipopolysaccharide neutralization, antigen presenting cell activity enhancement, signaling of epithelial cell apoptosis, and anti-neoplastic properties [10–14]. Vitamin D has an important role in the production of cathelicidin, directly increasing cathelicidin gene expression in a steroidal fashion [15]. Infection and chronic inflammation can also alter the process by which vitamin D promotes cathelicidin production [16, 17]. Cathelicidin plays an important role in innate immunity in the airway against both bacterial and viral pathogens. Cultured epithelium in smokers shows less secreted cathelicidin and decreased antimicrobial activity in response to Proteobacteria [18, 19]. Cathelicidin has also been demonstrated to directly perforate the viral envelope of respiratory syncytial virus and other important viral pathogens in respiratory tract infections [20, 21]. Cathelicidin levels can be measured in the sputum or plasma[22] and have been shown to respond to airway microbiologic burden[23], suggesting plasma cathelicidin levels may be indicative of the inflammatory state in the airways. In cross-sectional analysis, low plasma cathelicidin levels have been shown to relate to lower forced expiratory volume in 1 second (FEV1) and increased prevalence of pneumonia in individuals with or at-risk for COPD [24]. To date, no study has evaluated the association between cathelicidin measurements and longitudinal lung function changes in a well-characterized cohort of current or former smokers at risk for COPD.

The Study of HIV Infection in the Etiology of Lung Disease (SHIELD) is an ongoing prospective, observational cohort study of current and former injection drug users with and without HIV infection recruited in Baltimore, MD. Using longitudinal biological and pulmonary measures from the HIV-uninfected current or former smokers without spirometric evidence of COPD, we sought to determine the independent correlation between low cathelicidin levels and reduced lung function in a cohort at-risk for but without airflow obstruction. We also characterized the change in plasma cathelicidin measurements over five years. We hypothesized that low plasma cathelicidin levels would be independently associated with reduced FEV1 over time.

## Methods

### Study population

The SHIELD study follows a prospective cohort of predominantly African-American current or former injection drug users with prevalent tobacco use and vitamin D deficiency. The SHIELD study has followed participants in Baltimore, MD with twice yearly study visits along with pre-bronchodilator spirometry since 2007. The analytical cohort for this study included 308 HIV-seronegative current or former smokers with normal baseline spirometry, who had repeated spirometry testing and available samples for measure of plasma cathelicidin and serum 25-hydroxy-vitamin D level at baseline, year three, and year five of follow-up. This study was approved by the IRB of the Johns Hopkins University Bloomberg School of Public Health. All participants provided written informed consent.

### Measurements

Demographic data including age, sex, and race were collected at baseline. Smoking and injection drug use history were determined by participant report, with current smoking status defined as tobacco use within six months of enrollment. HIV status used for study exclusion was determined by serological testing. Blood samples for cathelicidin and vitamin D were collected at baseline and at follow-up years three and five. Plasma cathelicidin levels were measured using a commercially available ELISA assay (Hycult Biotech Inc, ELISA). 25-hydroxy-vitamin D levels were measured using commercially available radioimmunoassay (iDS, Enzyme Immunoassay). Spirometry data was collected at the time of each blood collection, as well as six months (median, 6.2 months; IQR 5.99–6.25 months) and 18 months (median, 18.6 months; IQR 17.93–18.49 months) after each blood collection. For this study, SHIELD participants were required to have blood and spirometry data at each time point and no evidence of obstructive lung disease at baseline visit, defined as pre-bronchodilator forced-expiratory-volume-in-one-second to forced vital capacity ratio (FEV1/FVC) >0.70.

### Statistical methods

Participants were stratified by baseline cathelicidin into lowest quartile (<35 ng/ml; n = 50) and higher cathelicidin levels (≥35 ng/ml; n = 258). Chi-squared testing, t-test (for normally distributed data), or the Kruskal-Wallis test (for skewed data) were used to determine the differences in demographic and clinical factors between the two cathelicidin groups and were reported using medians with interquartile ranges. Selected demographic and clinical factors (exposures) and FEV1 (outcome) were compared using univariate and multivariable logistic regression. Separate models evaluated associations with FEV1 at baseline, six and 18 months following cathelicidin measurement. Generalized estimating equations were used to account for repeated cathelicidin measurements during follow-up. Covariates retained in multivariate models were informed based on established clinical relevance and associations observed in univariate models, and included age, sex, race, current smoking status, and pack-years smoked. Stability of cathelicidin over time was graphically displayed with box-whisker plots and statistically assessed with paired t-tests. Sensitivity analyses were performed by adding height to the multivariable model assessing the independent effect of low cathelicidin on FEV1. The relationship between low cathelicidin and percent predicted FEV1 was assessed in a multivariable model with smoking history and current smoking as covariates. The correlation between vitamin D and cathelicidin was determined by Spearman correlation. For all statistical investigations, a P-value of <0.05 was considered statistically significant. All statistical analyses were performed using Stata version 13.1 (College Station, TX) and SAS version 12.1 (Cary, NC).

## Results

### Participant characteristics

The cohort of 308 participants was 91% African-American, with a mean age of 48.6 years, 32% female, 81% current smokers, with a mean pack-years smoked of 19 (IQR, 12.5–32 pack-years) (Table 1). At baseline, the median FEV1/FVC ratio was 0.79 (IQR, 0.76–0.83) with median baseline FEV1 of 2.98 L (IQR, 2.45–3.48 L) and median FEV1% predicted of 98% predicted (IQR, 88–108% predicted). The median plasma vitamin D level was 20.5 ng/ml (IQR, 14–27.1 ng/ml) and 48% of participants were vitamin D deficient (vitamin D <20 ng/ml) at baseline. The median plasma cathelicidin level was 52.3 ng/ml (IQR, 39.2–67.3 ng/ml).

**Table 1:** Baseline demographics of clinical cohort.

	Total	Low cathelicidin	High cathelicidin
N	308	50	258
Age, years	48.6 (43.7–52.9)	48.1 (44.12–51.7)	48.8 (43.6–53.5)
Female Sex	100 (32)	25 (50)	75 (29)
African-American	279 (91)	47 (94)	232 (90)
Current Smoker	250 (81)	43 (86)	207 (80)
Pack-years	19 (12.5–32)	17 (10.5–26.3)	19.1 (12.6–33)
BMI, kg/m^2^	25.8 (22.9–29.8)	26.5 (22.6–32.1)	25.8 (22.9–29.7)
Current Injection Drug Use	134 (43.5)	22 (44)	112 (43.4)
Annual Income <$5,000	228 (74.5)	42 (84)	186 (72.9)
FEV1/FVC	0.79 (0.76–0.83)	0.80 (0.75–0.83)	0.79 (0.76–0.82)
Absolute FEV1, liters	2.98 (2.45–3.48)	2.61 (2.3–3.3)	3.03 (2.50–3.52)
% Predicted FEV1	98 (88–108)	96 (87–105)	98 (88–109)
Vitamin D, ng/ml	20.5 (14–27.1)	17.3 (12.6–23.4)	20.9 (14.6–27.3)
Vitamin D <20 ng/ml	146 (48)	29 (58)	117 (46)
Cathelicidin, ng/ml	52.3 (39.2–67.3)	31.1 (26.5–33.3)	55.8 (45.7–72.8)

All values median (IQR) or n (%). Abbreviations: BMI (body mass index), FEV1 (forced expiratory volume in one second), FVC (forced vital capacity)

Median cathelicidin measurement was 31.1 ng/ml (IQR, 26.5–33.3 ng/ml) in the low cathelicidin cohort and 55.8 ng/ml (IQR, 45.7–72.8 ng/ml) in the high cathelicidin group (p<0.001). Compared with the high cathelicidin participants, those with low cathelicidin cohort were more likely to be female (50% vs. 29%; p = 0.004) and have a lower absolute FEV1 (2.61 L vs. 3.03 L; p = 0.008) (Table 1). There was no difference in cathelicidin levels comparing current to former smokers. Specifically, the median cathelicidin was 52.2 ng/ml (IQR, 39.1–67.4 ng/ml) among current smokers versus 53.6 ng/ml (IQR, 39.3–66.9 ng/ml) among former smokers (p = 0.72). Of current smokers, 17% had low cathelicidin compared with 12% of former smokers (p = 0.34). There was no difference in age, race, smoking status or intensity, percent predicted FEV1, injection drug use history, income, or vitamin D level between low and high cathelicidin groups.

### Cathelicidin trend in cohort

**Fig 1:**
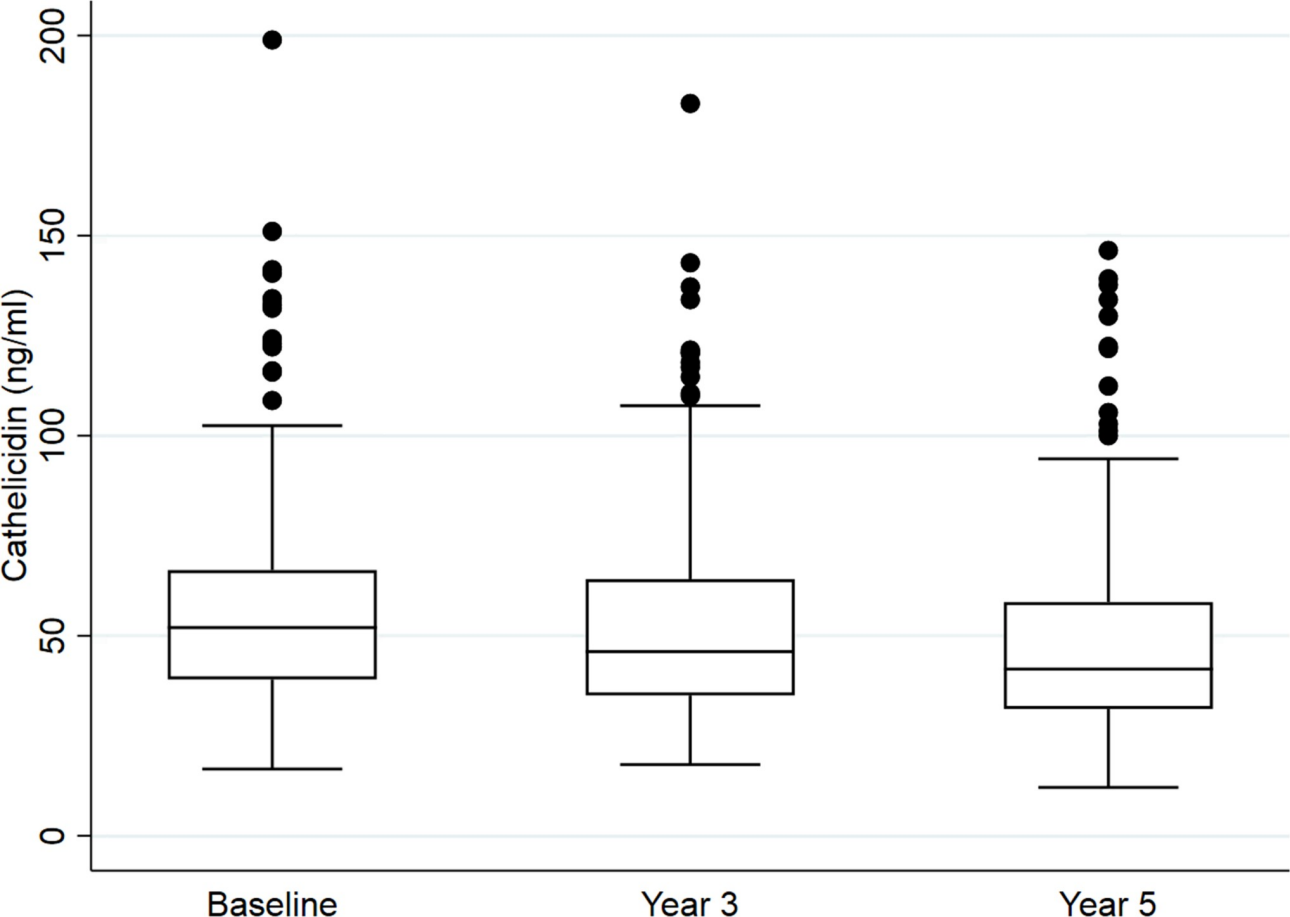
Cathelicidin measurements in cohort at years 1, 3, and 5. Repeated measurement of cathelicidin over time. Cathelicidin was stable between years one and three (p = 0.71). Year five measurements were lower than year one (p<0.001) and year three (p = 0.03).

Cathelicidin was stable between years one and three samples, but a small but statistical reduction was seen when comparing year one and year three to the year five samples (Fig 1). Specifically, there was no significant change between year one and year three cathelicidin measurements [median change, -4 ng/ml (IQR, -14.5 to 3.8 ng/ml); p = 0.71]. The change between cathelicidin levels measured at years three and five was quantitatively similar yet met statistical threshold of significance [median change, -4 ng/ml (IQR, -14.5 to 6.2 ng/ml); p = 0.03]. The change from year one to year five was also statistically different [median change, -8.7 ng/ml (IQR, -19.8 to 3.5 ng/ml); p<0.001].

### Relationship between cathelicidin and lung function

**Fig 2:**
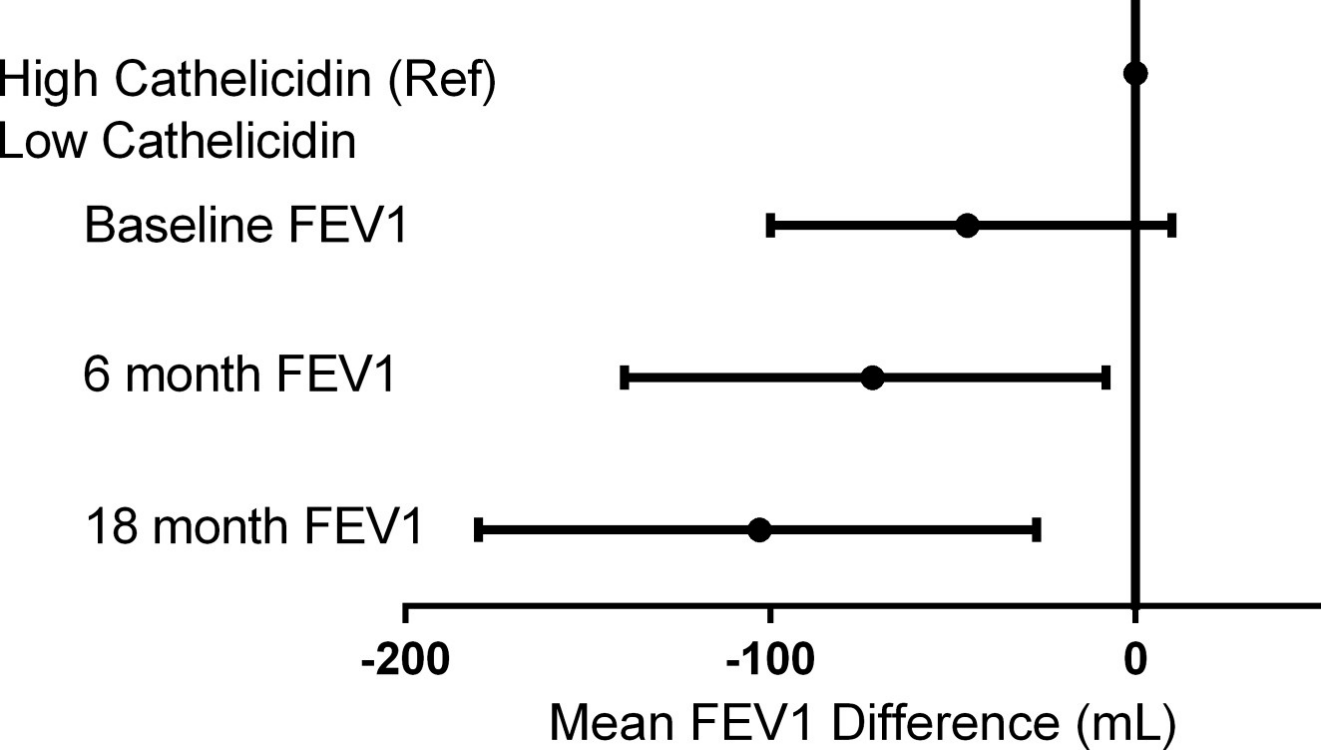
Adjusted association between cathelicidin and absolute FEV1 at baseline, 6 months, and 18 months. Low cathelicidin defined as <35 ng/ml. Values adjusted for age, race, sex, pack-years smoked, and current smoking status. Width of line represents 95% confidence interval.

In univariate analysis, several characteristics were associated with reduced FEV1 at time of cathelicidin measurement. For each 5-year increase in age, FEV1 was 270 ml lower (95% CI, -300 to -241 ml; p<0.001). African-American race was associated with 611 ml lower FEV1 (95% CI, -867 to -356 ml; p<0.001). Female sex was associated with 913 ml lower FEV1 (95% CI, -1043 to -782 ml; p<0.001). In this cohort with prevalent smoking and high smoking burden, current smoking status and pack-years smoked were not associated with lower FEV1 in univariate analysis. The lowest quartile of cathelicidin was associated with 93 ml lower FEV1 (95% CI, -210 to -160 ml; p<0.001) when compared to the higher cathelicidin group. In multivariate analysis including age, race, sex, current smoking status, and smoking pack-years, lower cathelicidin was not associated with significantly lower FEV1 at time of cathelicidin measurement [-46 ml (95% CI, -100 to 10 ml); p = 0.09] (Table 2, Fig 2). Other factors remained statistically associated with reduced FEV1 at time of cathelicidin measurement. These included age [-260 ml per 5 years of age (95% CI, -290 to -230 ml); p<0.001] and female sex [-1020 ml (95% CI, -1150 to -900 ml); p<0.001].

**Table 2:** Independent association between cohort characteristics and baseline FEV1*.

	FEV1, ml (95% CI)	P-value
Cathelicidin <35 ng/ml	-46 (-100 to 10)	0.09
Age, per 5 years	-260 (-290 to -230)	<0.001
African-American Race	10 (-200 to 210)	0.93
Female Sex	-1020 (-1150 to -900)	<0.001
Pack-years smoked, per pack-year	2 (-1 to 10)	0.23
Current Smoker	3 (-70 to 70)	0.92

*Model adjusted for all variables in table. Abbreviations: FEV1 (forced expiratory volume in one second)

For FEV1 obtained six months after baseline cathelicidin measurement, several characteristics were associated with lower FEV1 in univariate analysis. For each 5-year increase in age, six-month FEV1 was 188 ml lower (95% CI, -224 to -151 ml; p<0.001). African-American race was associated with 583 ml lower FEV1 (95% CI, -863 to -329 ml; p<0.001). Female sex was associated with 920 ml lower FEV1 (95% CI, -1049 to -791 ml; p<0.001). Current smoking status and pack-years smoked was not associated with a lower 6-month FEV1 in univariate analysis. Lowest quartile of cathelicidin was associated with 148 ml lower FEV1 at six months (95% CI, -220 to -70 ml; p<0.001) in univariate analysis. In multivariate analysis including age, race, sex, smoking status, and smoking pack-years, participants with low cathelicidin had 72 ml lower six-month FEV1 than those with higher cathelicidin (95% CI, -140 to -10 ml; p = 0.027) (Table 3, Fig 2). Other factors were associated with reduced six-month FEV1. These included age [-190 ml per 5-year increase in age (95% CI, -220 to -160 ml); p<0.001) and female sex [-1000 ml (95% CI, -1100 to -880 ml); p<0.001].

**Table 3:** Independent association between cohort characteristics and 6 and 18-month FEV1*.

	Six month		18 month	
	FEV1, ml (95% CI)	P-value	FEV1, ml (95% CI)	P-value
Cathelicidin <35 ng/ml	-72 (-140 to -8)	0.027	-103 (-108 to -27)	0.007
Age, per 5 years	-190 (-220 to -160)	<0.001	-170 (-200–130)	<0.001
African-American Race	-90 (-290 to 100)	0.36	-150 (-360 to 60)	0.15
Female Sex	-1000 (-1100 to -880)	<0.001	-960 (-1100 to -840)	<0.001
Pack-years smoked, per pack-year	1 (-2 to 4)	0.54	0.1 (-4 to 3)	0.97
Current Smoker	-30 (-110 to 50)	0.52	14 (-70 to 100)	0.75

*Model adjusted for all variables in table. Abbreviations: FEV1 (forced expiratory volume in one second)

At 18 months after each cathelicidin measurement, each 5-year increase in age was associated with a 177 ml lower FEV1 (95% CI, -214 to -139 ml; p<0.001). African-American race was associated with 610 ml lower 18-month FEV1 (95% CI, -876 to -358 ml; p<0.001). Female sex was associated with 894 ml lower FEV1 (95% CI, -1034 to -755 ml; p<0.001). Current smoking and pack-years smoked were not associated with lower 18-month FEV1 in univariate analysis. At 18 months after cathelicidin measurement, low cathelicidin was associated with 179 ml lower FEV1 in univariate analysis (95% CI, -254 to -104 ml; p<0.001). In multivariate analysis, low cathelicidin was associated with 103 ml lower FEV1 at 18 months (95% CI, -180 to -27 ml; p = 0.007) (Table 3, Fig 2). Other factors were associated with reduced 18-month FEV1. These include age [-170 ml per 5 years of age (95% CI, -200 to -130 ml); p<0.001) and female sex [-960 ml (95% CI, -1100 to -840 ml); p<0.001].

Due to the association between female sex and FEV in the multivariate modeling, the interaction between female sex and cathelicidin was investigated at baseline, six months, and 18 months. There was no interaction between low cathelicidin and female sex on FEV1 at time of cathelicidin measurement, (p = 0.65), six months after measurement (p = 0.54), or 18 months after measurement (p = 0.6).

### Sensitivity analyses of cathelicidin and FEV1 relationship

The addition of height to the multivariable models assessing the relationship between low cathelicidin and FEV1 did not attenuate effect at 6 months [-70.6 ml (95% CI -133 to -7.9); p = 0.027] or 18 months [-105 ml (95% CI -182 ml to -28.7 ml); p = 0.007]. In multivariable analysis controlling for smoking history and current smoking status, low cathelicidin was associated with a significantly decreased FEV1%-predicted at baseline [-3.40% (95% CI -5.37% to -1.43%); p = 0.001], 6 months [-2.77% (05% CI -4.86% to -0.67%); p = 0.01], and 18 months [-4.27% (95% CI -6.88% to -1.66%); p = 0.001].

### Cathelicidin and vitamin D

Across the cohort, serum vitamin D and plasma cathelicidin levels were not correlated (ϱ = -0.03; p = 0.40). Vitamin D levels were not associated with low cathelicidin when modeled continuously. Specifically, for each one ng/ml increase in vitamin D, cathelicidin increased 0.002 ng/ml (95% CI, -0.0004 to 0.0045 ng/ml; p = 0.11). A similar lack of association between vitamin D level and cathelicidin was seen when modeling vitamin D categorically (median cathelicidin: 52.8 ng/ml for those with vitamin D <10 ng/ml vs. 47.8 ng/ml for those with vitamin D from 10–20 ng/ml vs. 46.6 ng/ml for those with vitamin D ≥20 ng/ml; p = 0.34).

Compared to males, female participants had a significantly lower vitamin D [median 19.7 ng/ml (IQR 13.2 to 27.9)] vs. [median 21.4 ng/ml (IQR, 15.2–30.4)](p = 0.02). Stratified by sex, there was no association between vitamin D and cathelicidin among women (ϱ = -0.04; p = 0.52) or men (ϱ = -0.03; p = 0.47). By sex, the only association between vitamin D and cathelicidin <35 ng/ml was seen in women with vitamin D between 21–29 ng/ml [OR -0.13 (95% CI, -0.23 to -0.02); p = 0.024]. When analysis was restricted for male participants, vitamin D levels nor deficiency were not associated with odds of having a cathelicidin level <35 ng/ml, when modeled continuously or categorically.

## Discussion

To our knowledge, this is the first study to examine the longitudinal association between cathelicidin and subsequent FEV1 measured at six and 18 months of follow-up. We found that, in predominantly African-American current and former smokers without COPD, low plasma cathelicidin was associated with reduced lung function at six months and 18 months independent of age, sex, race, cumulative lifetime smoking, and current smoking status. Cathelicidin levels were largely stable over time, and we observed no correlation between serum vitamin D and plasma cathelicidin levels in this cohort with prevalent vitamin D deficiency. The results of this study suggest that reduced plasma cathelicidin may be an informative plasma biomarker to predict future lung function derangements in current and former smokers at risk for COPD.

Cross-sectional analysis of HIV-infected and HIV-uninfected individuals with or at-risk for obstructive lung disease demonstrated that low cathelicidin (<29 ng/ml) was associated with a 115 ml lower FEV1 at baseline when controlled for demographics, BMI, and smoking [24]. Our study refines and extends these observations by examining the longitudinal associations of cathelicidin and lung function in a cohort free of HIV infection. We show that cathelicidin is independently associated with six-month and 18-month reductions in FEV1 among smokers without COPD, ranging from 70–100 ml reductions in FEV1 with low cathelicidin. For comparison, an FEV1 difference of 100–140 ml represents the minimally important clinical difference for FEV1 in COPD pharmacological trials [25]. Unlike the prior cross-sectional study of cathelicidin in the SHIELD cohort[24], we did not observe a statistical association between cathelicidin and baseline FEV1, although the magnitude of baseline FEV1 reduction was similar. These differences may have arisen because our study only included HIV-uninfected individuals without COPD or that our study was a subset of the original study leading to underpowering to observe a difference.

The mechanisms underlying the association between low cathelicidin and FEV1 reductions cannot be fully evaluated in this observational cohort study. Cathelicidin may prevent lung function impairments by prevention of infection both directly and through relationships with immune cells[20, 21, 24, 26–29], and by interactions with regeneration and remodeling of epithelium [30, 31]. Recurrent infections are a known cause of FEV1 decline[32], and low cathelicidin plasma levels have been shown to portend to increased infections [24]. The innate immune system plays an important role to prevent pulmonary infections, bacterial colonization, and superinfection[23, 33–35], of which cathelicidin is an integral part. Measurable deficiency in cathelicidin in a cohort of current and former smokers may reflect decreased innate immune defense leading to frequent or chronic airway infection and FEV1 reduction.

This study did not show a correlation between plasma cathelicidin levels and serum levels of 25-hydroxy vitamin D. Vitamin D plays an important role in the immune system of the lungs via effects on promotion of the innate immune system including increasing the transcription of the gene encoding for cathelicidin, macrophage differentiation, signaling of cell apoptosis, modulating cellular adhesion, and by shunting the adaptive immune response in the lungs to less inflammatory pathways [36–40]. Increase in cathelicidin activity has been described in response to vitamin D supplementation[41], while other studies showed an inconclusive or absent association between serum vitamin D and cathelicidin levels [42–44]. Analysis of cultured cells has shown increased conversion of 25-OH-vitaminD to 1,25-OH-vitamin D by airway epithelia and upregulation of cathelicidin and other vitamin D receptor-mediated under direct exposure to 25-OH-vitamin D[45–47]. These findings suggest oral vitamin D supplementation and subsequent increased serum levels may not lead to increased vitamin D activity in airway epithelial cells potentially due to renal involvement in intermediary steps to conversion of bioactive 1,25-OH-vitamin D. Further, the lack of strong correlation between serum vitamin D levels and cathelicidin in our study may be due to the immunologic function of 1,25-hydroxy vitamin D (including increasing the expression of the cathelicidin gene) being dependent on local immune cells amplifying 1,25-hydroxy vitamin D concentrations via paracrine and autocrine signaling [48, 49]. While sufficient 25-hydroxy vitamin D is needed for this process, the intervening autocrine and paracrine step may not allow for a direct measurable correlation between 25-hydroxy vitamin D and cathelicidin levels. Further, this cohort had a high rate of current smokers and chemicals in cigarette smoke promote the expression of 24-hydroxylase in macrophages and block vitamin D-receptor-promoted transcription of the cathelicidin gene. This may result in disconnect between 25-hydroxy vitamin D levels and plasma cathelicidin levels [50]. Also, cigarette smoke reduces the microbial-killing activities of cathelicidin which may alter serum levels [18, 19]. These mechanisms highlight the complicated relationship between cathelicidin, vitamin D, and smoking. While we did not see a difference in cathelicidin levels between current and former smokers, the cohort is largely current smokers limiting the ability to further explore these relationships. Vitamin D regulates the production of cathelicidin and the supplementation of very low vitamin D may be of clinical importance, however, the complexity of the cathelicidin production pathway could make correlations between 25-hydroxy vitamin D and cathelicidin difficult to observe *in vivo*. The ability to augment cathelicidin levels by vitamin D supplementation has been demonstrated in select patient populations[28, 51, 52], with studies in smokers at-risk for COPD ongoing [53].

Female sex was associated with reduced cathelicidin. To our knowledge, this finding has not been reported previously. Despite this, a combination of female sex and low cathelicidin was not found to be associated with longitudinal FEV1 reductions. The interaction between cathelicidin and female sex has been investigated in other disease states. Female fetuses are shown to have a lower risk of perinatal infections thought to be due to testosterone inhibition of vitamin D upregulation of placental cathelicidin in male fetuses [54]. In adult females, reduced cathelicidin is associated with bacterial vaginosis[55], rosacea[56], and recurrent urinary tract infections [57]. When combined with the findings presented here, the role of hormonal changes on cathelicidin levels may offer insight into altered immunologic function which deserves future studies.

Longitudinal studies suggest that early life factors in smokers including crowded living environments, working-class social status, early childhood respiratory infections, low childhood lung function, male sex, and childhood asthma contribute to the development of obstructive lung disease at a relatively young age [5, 9, 58, 59]. Small airway disease on computed tomography imaging is associated with an increased rate of FEV1 decline in adult smokers, as well [60, 61]. Further, non-specific markers of inflammation, individually and in combination, have been associated with increase in the rate of FEV1 decline and progression of emphysema across two cohorts [62]. Determining the independent association between plasma cathelicidin, an important component in the innate defense of the lung, and poorer longitudinal lung function in at-risk individuals raises the possibility of predicting future lung function impairments based on a single blood test, enhancing the above findings.

This investigation has limitations. These observations are evaluated in only a single cohort of predominantly African-American participants, which limits generalizability. However, the high rate of current and former smokers at risk for vitamin D deficiency make this cohort potentially more likely to be affected by low cathelicidin, and, thus, an ideal population to study these research questions. Greater variation in the race and locale of participants would allow for more variability in vitamin D levels. Concomitant Western blot testing has been used in tandem with ELISA measurements [63, 64] and would have added robustness to analysis. Also, analysis of other defensins active in the innate immune system would add information to the results of this study. The presence of pneumonia data is lacking in this study and would allow a more robust assessment of the hypothesis that the mechanism of the low cathelicidin and reduced lung function is due to frequent infection. Presence of respiratory exacerbation and imaging data would further enhance findings in this cohort.

In conclusion, in a cohort of predominantly African-American current and former smokers, low plasma cathelicidin was associated with a significantly lower FEV1 at six and 18 months of observation. This relationship was independent of demographic factors and smoking history. Cathelicidin was shown to be largely stable over time, highlighting its potential as an informative biomarker. There was a decrease in cathelicidin level at year 5 which may limit use of cathelicidin in long term lung function outcomes. It is possible that the increased risk of airway infection with low cathelicidin leads to the observed decrease in FEV1, and that determining cathelicidin levels in smokers with preserved lung function can predict the likelihood of impaired long-term lung function. Further, early detection and augmentation of cathelicidin levels may constitute a therapeutic approach to smokers at risk for developing obstructive lung disease.
